# Associations Between Glutathione-S-Transferase Genotypes and Bronchial Hyperreactivity Patients With Di-isocyanate Induced Asthma. A Follow-Up Study

**DOI:** 10.3389/fmed.2019.00220

**Published:** 2019-10-09

**Authors:** Jussi Leppilahti, Marja-Leena Majuri, Timo Sorsa, Ari Hirvonen, Päivi Piirilä

**Affiliations:** ^1^Department of Periodontology and Geriatric Dentistry, University of Oulu, Oulu, Finland; ^2^Oulu University Hospital, Oulu, Finland; ^3^Finnish Institute of Occupational Health, Helsinki University, Helsinki, Finland; ^4^Department of Oral and Maxillofacial Diseases, University of Helsinki, Helsinki, Finland; ^5^Department of Oral and Maxillofacial Diseases, Helsinki University Central Hospital, Helsinki, Finland; ^6^Division of Oral Diseases, Department of Dental Medicine, Karolinska Institute, Huddinge, Sweden; ^7^National Supervisory Authority for Welfare and Health, Valvira, Helsinki, Finland; ^8^Unit of Clinical Physiology, HUS Medical Imaging Center, Helsinki University Central Hospital, University of Helsinki, Helsinki, Finland

**Keywords:** N-acetyl transpherases, oxidative stress, enzyme activity, GSTP1 Val105/Val105, genetic polymorphism, GSTM1, GSTT1, occupational asthma

## Abstract

**Introduction:** Di-isocyanates TDI (toluene di-isocyanate), MDI (diphenylmethane di-isocyanate), and HDI (hexamethylene di-isocyanate) are the most common chemicals causing occupational asthma. Di-isocyanate inhalation has been reported to induce oxidative stress via reactive oxygen and nitrogen species leading to tissue injury. Glutathione transferases (GSTs) and N-acetyltransferases (NATs) are detoxifying enzymes whose general function is to inactivate electrophilic substances. The most important genes regulating these enzymes, i.e., *GSTM1, GSTP1, GSTT1, NAT1*, and *NAT2* have polymorphic variants resulting in enhanced or lowered enzyme activities. Since inability to detoxify harmful oxidants can lead to inflammatory processes involving activation of bronchoconstrictive mechanisms, we studied whether the altered *GST* and *NAT* genotypes were associated with bronchial hyperreactivity (BHR) in patients with di-isocyanate exposure related occupational asthma, irrespective of cessation of di-isocyanate exposure, and adequacy of asthma treatment.

**Methods:** Polymerase chain reaction (PCR) based methods were used to analyze nine common polymorphisms in *GSTM1, GSTM3, GSTP1, GSTT1, NAT1*, and *NAT2* genes in 108 patients with diagnosed occupational di-isocyanate-induced asthma. The genotype data were compared with spirometric lung function and BHR status at diagnosis and in the follow-up examination on average 11 years (range 1–22 years) after the asthma diagnosis. Serum IgE and IL13 levels were also assessed in the follow-up phase.

**Results:** An association between BHR and *GSTP1* slow activity (Val105/Val105) genotype was demonstrated in the subjects at the follow-up phase but not at the diagnosis phase. Moreover, the patients with the *GSTP1* slow activity genotype exhibited characteristics of Th-2 type immune response more often compared to those with the unaltered *GSTP1* gene. Interestingly, all 10 patients with the *GSTP1* slow activity genotype had both the *GSTM3* slow activity genotype and the unaltered *GSTT1* gene.

**Discussion:** The results suggest associations of the low activity variants of the *GSTP1* gene with BHR. The fact that these associations came up only at the follow-up phase when the subjects were not any more exposed to di-isocyanates, and used asthma medication, suggest that medication and environmental factors influence the presentation of these associations. However, due to the exploratory character of the study and relatively small study size, the findings remain to be confirmed in future studies with larger sample sizes.

## Introduction

Di-isocyanates TDI (toluene di-isocyanate), MDI (diphenylmethane di-isocyanate), and HDI (hexamethylene di-isocyanate), generally used in the manufacture of plastics, paints, and foam products, have long been the most common chemicals causing occupational asthma. Bronchial inflammation, including lymphocytic infiltration and eosinophilia (1–3), resembling that present in allergic asthma, has been demonstrated to be associated with di-isocyanate-induced asthma (DIA). After cessation of exposure, suggestion of a reversal of remodeling of the airway wall has been found (4, 5) but respiratory symptoms (6, 7) and nonspecific bronchial hyperreactivity (BHR) have been reported to persist for years in the majority of patients with DIA despite cessation of exposure and asthma treatment (8–11). Treatment with inhaled steroids causes a decrease in BHR (9, 12, 13).

Inhalation of di-isocyanates have in several studies been found to induce oxidative stress via reactive oxygen and nitrogen species causing tissue injury (14–19). Glutathione *S*-transferases (GSTs) form a family of detoxifying enzymes whose general function is to inactivate electrophilic molecules. GSTs detoxify those substances by glutathione conjugation, and therefore a variety of harmful agents, e.g., products of reactive oxygen species or exogenous electrophilic substances may act as substrates for GSTs and become detoxified (20). Inability to detoxify products of oxidative stress could eventually lead to inflammatory process and activation of bronchoconstrictive mechanisms, and ultimately to the development of asthma.

The most important *GST* genes, i.e., *GSTM1, GSTM3, GSTP1*, and *GSTT1* are polymorphic exhibiting variants associated with an altered enzyme activity. Other polymorphic enzymes involved in the defense against reactive metabolites include *N*-acetyltransferases (NAT) being involved in the deactivation of aromatic amines in degradation of aromatic di-isocyanates. Their genes, *NAT1* and *NAT2*, have also been shown to exhibit polymorphic variants resulting in altered enzyme activities (21).

In occupational asthma, polymorphic GSTP1 enzyme, especially its slow activity genotype, has been widely studied as a potential candidate to lowered protection against oxidative burden. The *GSTP1* slow activity genotype has been found mainly to be protective for the development of asthma (22, 23). However, for example in a study on association of traffic-related air pollution with asthma, the *GSTP1* slow activity genotype, as well as the *GSTT1* null genotype, were found to be associated with increased risk for asthma, wheeze, and lowered lung function (24). Numerous studies have also been seeking the associations between the polymorphic antioxidant enzymes and occupational asthma or BHR. Some studies (22, 23, 25) have found *GSTP1* slow activity variant and *GSTM1* null genotype to decrease the risk for DIA (25), while others have found *GSTP1* low activity genotypes to increase IgE-mediated reactions in DIA (26, 27).

We have earlier observed in a study population of 182 workers exposed to di-isocyanates, 109 of whom had earlier been diagnosed with asthma, that genetic factors, especially the *GSTM3* and *NAT2* genotypes, modified risk for occupational asthma and allergic responses to di-isocyanate exposure (26, 28). These studies were performed when the examined patients were called for clinical control on average 11 years (range 1–22 years) after diagnosis. However, the risk for occupational asthma (26, 28) was analyzed with data from the diagnosis phase, concentrating with, e.g., the specific challenge test results, the presence of non-specific bronchial challenge or specific IgE in the diagnostic baseline examinations. The lung function data from the follow-up visit was published separately (11) and was not analyzed in relation to the *GST* or *NAT* genotype data.

The aim of the present study was to examine the possible effects of altered activity associated *GST* and *NAT* genotypes on the risk of BHR several years after cessation of di-isocyanate exposure when most of the study subjects were not any more occupationally exposed to di-isocyanates and were on asthma medication if their condition required it.

Two earlier enrolled study populations with follow-up examinations (11, 13, 26, 28) were available for the present study. In addition, analysis of cytokines and chemokines was performed from the blood specimen available. The working hypothesis was that the reduced activity variants of the polymorphic enzyme genes or their combinations would cause a poor outcome measured as presence of non-specific BHR.

## Materials and Methods

### Study Subjects

The study subjects (*N* = 108) were pooled from two earlier studies; most of the subjects (*n* = 93) were from the study described in Piirilä et al. (26) and Wikman et al. (28), the remainder (*n* = 15) were included in another study carried out by Piirilä et al. (13). All subjects were studied at the Finnish Institute of Occupational Health (FIOH) and diagnosed to have di-isocyanate-induced occupational asthma on average 11 years before follow-up examinations (Table 1). For most of the patients, the di-isocyanate exposure had ceased after the diagnosis of occupational asthma. For six patients, minimal exposure to di-isocyanates was still possible in the factories, although it was not anymore possible in their own working points. The occupational asthma diagnoses were made in 1976–1999 and the follow-up examinations were performed in 1995–2001.

**Table 1:** Anthropometric data, smoking, exposure, hyperreactivity, and diagnostic criteria of occupational asthma of the patients.

		**Occupational diagnosis phase**	**Follow-up phase**
Gender	Male	84 (78%)	84 (78 %)
	Female	24 (22%)	24 (22%)
Age (years)		39 (21–60)	50 (48–52) (26–78)
Length of follow-up (years)			11.3 (1–22.0)
Weight (kg)		79.4 (51–132)	
Height (cm)		173 (153–191)	
BMI		26.6 (18.7–41.2)	
Smoking	No	56 (52%)	
	Yes	33 (31%)	
	Former	19 (18%)	
Smoking pack—years	Current	15.8 (0.3–51.3)	
	Former	9.5 (0.1–28)	
DIA exposure	TDI	25 (23%)	
	MDI	45 (42%)	
	HDI	42 (39%)	
Basis of diagnosis of occupational asthma	Specific bronchial challenge testing	94 (87%)	
	PEF work place follow-up	14 (13%)	
Reaction in the specific challenge test at the diagnosis	Immediate	53 (49%)	
	Late	41 (38%)	
	Not tested	14 (13%)	
Continuing DIA exposure after occupational asthma diagnosis		6 (6%)	
BHR	Negative	51 (47%)	45 (42%)
	Positive	51 (47%)	60 (56%)
	Not tested	6 (6%)	3 (2%)
FEV1 (% of pred.)		99 (96–101)	91 (88–94)
VC (% of pred.)		99 (96–101)	97 (94–100)

### Methods

The methods for the occupational studies have been presented in Piirilä et al. (11) and Piirilä et al. (13), and the *GST* and *NAT* genotype analyses were conducted as described in Piirilä et al. (26) and Wikman et al. (28).

The flow-volume spirometry was performed with a rolling-seal spirometer (Mijnhardt BV, Bunnik, the Netherlands) connected to a microcomputer (Medikro MR-3; Medikro, Kuopio, Finland) and the results were compared with the reference values of Viljanen et al. (29).

The non-specific bronchial challenge tests were performed with the Sovijärvi method (30). FEV1 was measured with a Vitalograph S bellow spirometer (Vitalograph). 1.6% histamine diphosphate was used and a 15% reduction of FEV1 was the limit of significant reaction (Provocative dose, PD15). The hyperresponsiveness was graded as strong with PD15 < 0.1 mg, moderate with 0.11–0.4 mg, and mild with 0.41–1.6 mg provocative doses of histamine. The histamine challenge test was not performed if FEV1 was <70% of predicted value (30). In the present analyses, any degree of BHR was regarded as positive for BHR.

The cytokines and chemokine levels were analyzed with Luminex technology (Bio-Plex 200 System, Bio-Rad Laboratories, Hercules, CA, USA) by labeled cytokine capture antibody pairs from venous serum samples stored deep frozen in −70°C. In addition, the total IgE was determined with the earlier indicated methods (11).

Covariation between BHR at the time of diagnosis and the follow-up examination and different *GSTM1, GSTM3, GSTP1, GSTT1, NAT1*, and *NAT2* genotypes were first explored with cross-tabulations and Pearson chi-squared tests. Next, the effect of other putative predictive and modifying factors, age, gender, smoking, inhaled corticosteroid, or per oral cortisone treatment during the year preceding the follow-up examinations, regular NSAID usage (for other medical purposes), and hyper-reactivity at the time of diagnosis (in case of follow-up BHR), were taken into account in a multivariate logistic regression model.

The effect of *GST* and *NAT* genotypes on the follow-up FEV1% (age, sex and body composition adjusted) were analyzed by a generalized linear model (GLM) adjusted by putative other modifying factors; follow-up hyperreactivity, inhaled, or peroral corticosteroid treatment during the year preceding the follow-up examination, regular NSAID usage (for other medical purposes) and smoking.

The *GSTP1* genotype could not be included in the regression model due to the low number of the homozygous slow activity genotypes in the study population overall, and especially because there were no carriers of the slow activity genotype among the non-BHR patients. *Post-hoc* analysis of interactions between *GSTP1* and other *GST* and *NAT* genotypes and different medications were explored by cross-tabulation and Pearson chi-squared tests. In addition, IL-13 and total IgE levels were compared between *GSTP1* groups with the non-parametric Kruskal-Wallis method.

Mean and 95% confidence intervals were used with continuous variables in purpose to describe patient characteristics. In the statistical evaluations the limit of statistical significance was set to *p* < 0.05.

All statistical analyses were performed using the SPSS (version 24) statistical software.

## Results

### Association Between Bronchial Hyperreactivity and the GST and NAT Genotypes

Distribution of different *GST* and *NAT* genotypes in the study population is described in Table 2. Associations between *GST* and *NAT* genotypes and BHR at the diagnostic examinations of occupational asthma and at the follow-up examinations, explored in a cross-tabulation without the adjustments of other covariates are presented in Table 3. Statistically significant associations (*p* < 0.05) were observed between *GSTM1* and *GSTP1* genotypes and the BHR at the follow-up examination. Interestingly, all patients with homozygous *GSTP1* slow activity genotype (*N* = 10) had developed BHR.

**Table 2:** Distribution of the *NAT* and *GST* genotypes among the study subjects.

**Enzyme**	**Genotype**	***N* (%)**
NAT1	Slow activity	8 (8%)
	Intermediate activity	68 (65%)
	Fast activity	29 (28%)
NAT2	Low activity	53 (50%)
	High activity	52 (50%)
GSTM1	Null	44 (42%)
	Positive	58 (55%)
GSTM3	Low activity	83 (79%)
	High activity	22 (21%)
GSTP1	Homozygous fast activity	47 (45%)
	Heterozygous fast activity	48 (46%)
	Slow activity	10 (9.5%)
GSTT1	Null	12 (11%)
	Positive	93 (89%)

**Table 3:** Cross-tabulation of GST and NAT enzymes with bronchial hyperreactivity.

**Outcome of BHR**		**Occupational diagnosis phase**				**Follow-up phase**			
		**No (*n* = 38)**	**Yes (*n* = 53)**	**Not tested**	*p*-value^*^	**No (*n* = 51)**	**Yes (*n* = 49)**	**Not tested**	*p*-value^**^
GSTM1	Null (*n* = 58)	27	26	5	Ns	27	29	2	<0.05
	Positive (*n* = 47)	23	23	1	–	16	31	0	
GSTM3	Low activity (*n* = 83)	41	38	4	Ns	34	48	1	Ns
	High activity	9	11	2	–	9	12	1	
GSTP1	Slow activity	5	4	1	Ns	0	10	0	<0.05
	Fast activity#	45	45	5	–	43	50	2	
GSTT1	Null	5	7	0	Ns	4	8	0	Ns
	Positive#	45	42	6	–	39	52	2	
NAT1	Slow activity	4	4	0	Ns	4	3	1	Ns
	Intermediate activity	30	32	6	–	26	41	1	
	Fast activity	16	13	0	–	13	16	0	
NAT2	Slow activity	26	25	2	Ns	23	30	0	Ns
	Fast activity (wild type genotype)	24	24	4	–	20	30	2	

The p-values (Pearson chi-square test) indicate significant association of the genotypes with presence of bronchial hyperreactivity at the occupational diagnosis phase (^*^) and follow-up phase (^**^), #, positive homo- or heterozygote.

No significant associations were detected between the *GST* and *NAT* genotypes and BHR at the time of diagnosis. Moreover, there was no significant association between *GSTM1* genotype and presence of any level of BHR in a multivariate logistic regression model when the model was adjusted by the putative other risk factors.

In the logistic regression model, the presence of persistent BHR of patients was significantly (*p* < 0.05) associated with female gender, smoking at the occupational diagnosis phase, and previous BHR at the time of occupational diagnosis (Table 4). None of the studied covariates/cofactors (age, gender, smoking, and *GST* and *NAT* genotypes) were associated with the BHR at the time of occupational diagnosis. The *GSTP1* genotype could not be included in the regression model due to the low number of the homozygous slow activity genotypes in the study population overall, and especially because there were no carriers of the slow activity genotype among the non-BHR patients.

**Table 4:** The association between BHR and putative modifying factors in a logistic regression model (non-significant factors removed).

**OUTCOME: FOLLOW-UP BHR (*****n*** **=** **99)**		**OR (95%CI)**	**Significance of hypothesis test**
Previous BHR at the occupational diagnosis phase	Positive (*n* = 47)	4.6 (1.6; 13.3)	<0.05
	Not tested (*n* = 6)	10.4 (0.9; 115.9)	Ns
	Negative (*n* = 46)	1	
Gender	Female (*n* = 21)	8.1 (1.9; 34.5)	<0.05
	Male (*n* = 78)	1	–
Smoking at occupational diagnosis phase	Yes (*n* = 31)	4.8 (1.5; 15.8)	<0.05
	Former (*n* = 19)	2.5 (0.7; 8.7)	Ns
	No (*n* = 49)	1	
GSTM1	Positive (*n* = 45)	1.7 (0.6–4.5)	Ns
	Not known (*n* = 2)	1 (0–1)	Ns
	Null (*n* = 52)	1	
Age		1.06 (1.01–1.12)	<0.05

### Characteristics of Patients With the Homozygous GSTP1 Slow Activity Genotype

Of the patients with the *GSTP1* slow activity genotypes, three had been exposed to TDI, four to MDI, and one to HDI. All patients with *GSTP1* slow genotype were still working but were not anymore exposed to di-isocyanates at their work places. The mean follow-up duration after diagnosis phase was 11 (SD 5.1, range 5–19) years for the carriers of the *GSTP1* slow activity genotype, which was exactly the same as for the fast, homozygote or heterozygote genotype subjects (mean 11 years, SD 5.5 years, range 1–22 years).

Four of the patients with *GSTP1* slow genotype were nonsmokers, three former smokers (mean pack-years 4.2, range 0.5–10.5), and three current smokers (mean pack years 13, range 10–20) based on the occupational diagnosis phase. The patients with *GSTP1* slow activity genotype had smoked considerably less (mean pack-years 5.25, SD 7.0, range 0–20) than those with the fast activity genotype (mean pack-years 8.37, SD 11.9, range 0–51.3).

Gender was found to be associated with the BHR in the present study population; half of the patients with the *GSTP1* slow activity genotype were women, which exceeds the observed relative frequency of this genotype in men.

All the patients with *GSTP1* slow activity genotype were already using or were prescribed to start inhaled corticosteroid medication at the follow-up examinations. These patients also needed peroral corticosteroid medication more often during the year preceding the follow-up examinations (Table 5). However, no straightforward dose-response relationship was observed between the need for medication and different *GSTP1* genotypes (Table 5).

**Table 5:** Potential interacting factors, smoking, and medications of patients with different GSTP1 genotypes during the follow-up and interaction between *GSTP1* and other *GST* genes and *NAT* genes.

		**GSTP1 “slow”**	**GSTP1 “heterozygous fast”**	**GSTP1 “homozygous fast”**	**Total**	**Statistical significance**
**USE OF MEDICATION DURING FOLLOW UP AND SMOKING HABITS**						
Short-acting beta agonists	Yes	7	21	26	54	Ns
	No	3	27	21	51	
Inhaled steroids	Yes	7	12	20	39	<0.05
	No	3	36	27	66	
Long-acting beta agonists	Yes	1	5	5	11	Ns
	No	9	43	42	94	
Anti-inflammatory (NSAID) medication	Yes	2	9	12	23	Ns
	No	8	38	34	80	
Corticosteroid medication during the year preceding the follow-up	Yes	3	2	4	9	<0.05
	No	7	45	42	94	
Smoking at occupational diagnosis phase	Yes	3	14	15	32	Ns
	No	4	28	22	54	
	Former	3	6	10	19	
**INTERACTION OF GENOTYPES**						
GSTM1 genotypes	Null	7	28	23	58	Ns
	Positive (hetero- or homozygous)	3	20	24	47	
GSTM3 genotypes	Low activity	10	34	39	83	Ns
	High activity	0	14	8	22	
GSTT1 genotypes	Null	0	10	2	12	<0.05
	Positive (hetero- or homozygous)	10	38	45	93	
NAT1 genotypes	Low activity	0	4	4	8	Ns
	Intermediate activity	8	31	29	68	
	High activity	2	13	14	29	
NAT2 genotypes	Low	3	23	27	53	Ns
	High	7	25	20	52	

*The p-values (Pearson chi-square test) indicate differences in use of asthma medication and smoking (above) and interaction of the genotypes (lower)*.

**Figure 1:**
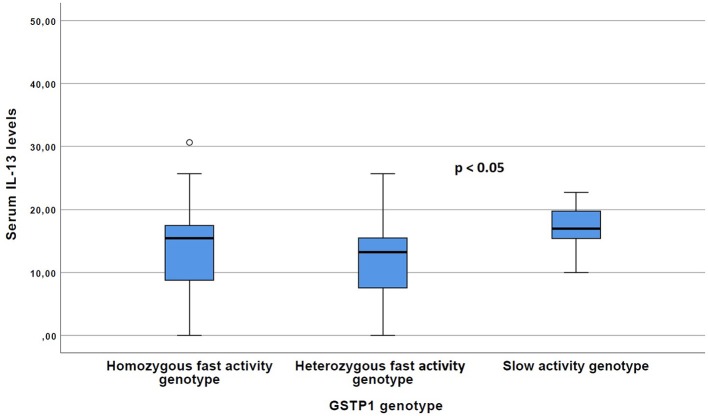
The Serum IL-13 levels of the patients with GSTP1 slow activity genotype compared with those with homozygous fast activity and heterozygous fast activity genotypes. Boxplots with median (line) and respective 25 and 75 percentiles as well as outliers with circles are given. *P*-value have been received with the Kruskal–Wallis test.

**Figure 2:**
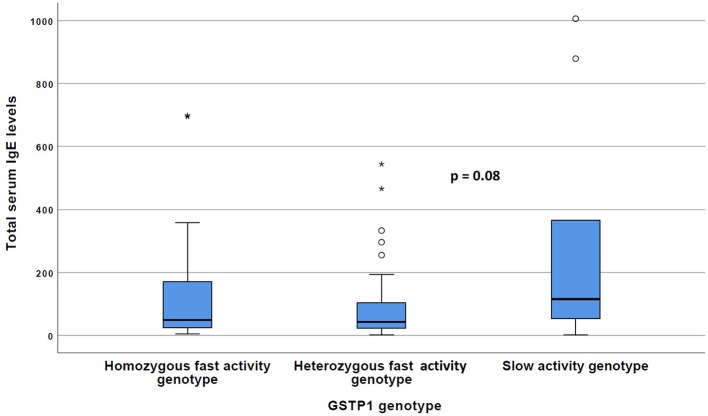
The total IgE levels of the patients with GSTP1 slow activity genotype compared with those with homozygous fast activity and heterozygous fast activity genotypes. Boxplots with median (line) and respective 25 and 75 percentiles are given, outliers indicated with circles and asterisks. *P*-value has been received with the Kruskal–Wallis test.

The carriers of the *GSTP1* slow activity genotype more often exhibited characteristics of Th2 type cytokine profile compared with the *GSTP1* fast activity genotypes. For example, serum IL-13 levels were significantly (*p* < 0.05) increased within these patients compared to the patients with the *GSTP1* fast activity genotypes (Figure 1), and there was a statistically non-significant (*p* = 0.08) trend for increased total serum IgE levels in these patients (Figure 2).

### Association Between FEV1% and GST and NAT Genotypes at the Follow-Up

When the association between FEV1 percent of predicted value (FEV1%) and the *GST* and *NAT* genotypes together with putative modifying factors were studied with the generalized linear model (Table 6), none of the studied genotypes were associated with FEV1% when studied separately. However, the homozygous *GSTP1* slow activity genotype seemed to modify the effect of BHR and inhaled corticosteroid medication on follow-up FEV1%. Paradoxically, the FEV1% values were better among *GSTP1* slow activity genotype carriers compared to patients with *GSTP1* fast activity genotype, when BHR and regular corticosteroid medication were taken into account in the analysis. At the time of diagnosis, there were no significant association between the studied genotypes and FEV1%.

**Table 6:** The effect of significant predictive factors on follow-up FEV1% in the regression model.

**OUTCOME: FEV1% AT FOLLOW-UP (*****n*** **=** **99)**		**Estimated effect size (95%CI)**	**Estimated marginal mean (95%CI)**	Significance of hypothesis test^*^
BHR	Yes (*n* = 58)	−13 (−19; −8)	89 (85; 93)	<0.001
	Not tested (*n* = 2)	−2 (−21;16)	100 (82; 118)	Ns
	No (*n* = 39)	0	102 (96; 108)	–
Inhaled glucocorticoid medication	Yes (*n* = 33)	−13 (−19; −8)	90 (83; 98)	<0.001
	No (*n* = 66)	0	104 (96; 112)	–
GSTP1	Fast activity (hetero-/homozygote) (*n* = 89)	−14 (−23; −5)	90 (84; 96)	<0.05
	Slow activity (*n* = 10)	0	104 (93; 114)	–

* *Bonferroni correction in multiple hypothesis testing*.

## Discussion

We demonstrated an association between non-specific BHR and the *GSTP1* slow activity genotype (Val105/Val105) in patients with DIA in the follow-up examinations on average 11 years after the diagnosis phase. In contrast, no associations could be observed between BHR and *GST* or *NAT* genotypes at the diagnosis phase.

At the follow-up phase, BHR also associated with the conventional risk-factors, smoking, gender, and previous BHR measured at the diagnosis phase. Moreover, at this phase the patients with the *GSTP1* slow activity genotype exhibited characteristics of Th-2 type immune response, and higher IL-13 and IgE levels compared to those with the *GSTP1* fast activity genotype. All 10 patients with the *GSTP1* slow activity genotype had BHR in follow-up examinations, which is unlike to occur by chance. All these patients also carried *GSTM3* low activity genotype and unaltered *GSTT1* gene. Furthermore, most (7 out of 10) of them had *GSTM1* null genotype (Table 5). This suggests an interaction between *GSTP1* and the other studied *GST* genes, affecting on the observed statistical association between the *GSTP1* genotype and BHR.

Kamada et al. (31) demonstrated an association of *GSTP1* slow activity genotype with childhood asthma. They also found *GSTM1* positive genotype with normal activity to modify this association. Interestingly, the present results also suggest an association of *GSTP1* and *GSTM1* genotypes with BHR. Differing from their results of the earlier study (31), most patients in the present study with *GSTP1* slow genotype concurrently carried the *GSTM1* null genotype. Moreover, in the present study all patients with *GSTP1* slow genotype showed normal *GSTT1* genotype. It is therefore tempting to speculate that the normally acting variant would participate in the development or pertinence of BHR. As the positive *GSTT1* genotype is rather common, however, reliable conclusions cannot be drawn from this association.

In contrast to the follow-up phase, we observed no significant association between the reduced activity associated genotypes and BHR at the diagnostic phase. Interestingly, in the follow-up phase, BHR and signs of inflammation in carriers of *GSTP1* slow activity genotype were present despite ongoing treatment with asthma medication, suggesting that chronic inflammation had developed in their bronchi more often than in subjects with unaltered activity associated *GSTP1* genotypes. This also suggests that the associations of the low activity *GST* variants with BHR or asthma cannot always be documented, but it can be seen when the conditions are proper to call these latent properties forth.

To date, a large number of studies have been conducted on non-occupational or occupational asthma, with varying results on the association of *GST* genotypes with risk of developing asthma or BHR (32–35). Increased risk between *GST* genotypes and development of asthma, BHR, wheezing or IgE mediated inflammation (24, 26, 27, 35) and even an additive effect of *GSTT1* and *GSTP1* low activity genotypes in the development of asthma have also been suggested (32). However, there are also numerous studies suggesting that *GSTP1* slow activity variant protects from developing asthma or BHR (22, 23, 25, 36–40), and several studies have found no association between the *GST* genotypes and BHR or asthma (33, 34, 41–43). The present results might offer an explanation at least to some of these variable results.

The prevalence of the low activity associated gene variants or distribution of the GST's in the lungs may also depend on ethnicity. In a meta-analysis, increased risk for asthma was found in the presence of the *GSTM1* and *GSTT1* low activity genotypes, the risk varying depending on ethnicity (35). One explanation for the possible ethnic differences could be dissimilar prevalence of detoxifying enzymes with low activity in different ethnic groups. For example, the prevalence of *GSTM1* null genotype has been found to vary between 26 and 48%, *GSTT1* null from 20 to 46%, the homozygous fast activity variant *GSTPI* Ile/Ile between 29 and 49%, and the slow activity Val105/Val105 genotype between 7 and 22% (44) in different ethnicities. Among the present study subjects, all of whom were of Finnish descent, the prevalence of the *GSTP1* fast activity and slow activity genotypes were about 45 and 10%, respectively, which are in line with previous findings in Finnish population (45).

Our patients with *GSTP1* slow activity genotype showed increased IL-13 levels, which is related to TH2-type cytokine profile suggesting permanent asthmatic inflammation in those with *GSTP1* slow genotype. In an earlier study, the *GSTP1* slow activity genotype was found to be associated with increase of several acute phase cytokines (TNF-a, IL-6, CXCL8, IL-12, CCL11, thromboxane B2) and immunoglobulin E when studied after specific allergen challenge, but no association between BHR and *GSTP1* genotype was observed (36). Also, association of *GSTP1* slow genotype with allergic asthma or IgE- mediated reactions have earlier been suggested (26, 34).

Environmental exposure, e.g., traffic-related air pollution, has been suggested to be involved in the development of bronchial inflammation leading to BHR or asthma in the presence of low activity enzyme variants of the detoxifying enzymes (24, 34). Also smoking of cigarettes causes increased exposure to, e.g., superoxide, hydrogen peroxide, nitric oxide or nitrites (46), which precipitates the development of bronchial inflammation leading to asthma or BHR in subjects with low activity variants of polymorphic detoxifying enzymes. In this study, a slight majority (60%) of the patients with *GSTP1* slow activity genotype were current or ex-smokers compared to the whole study population (33%) or to the fast genotype (normal and intermediate) patients (47%). However, those with normal enzyme activity had smoked more than those with the slow enzyme activity, although the difference did not reach statistical significance. The highest pack-years (up to 50) were found in the patients with the fast activity genotype. This suggests that the study subjects were similarly exposed to smoking, and therefore it is not probable that smoking would be a crucial reason for the slow activity genotype subjects to develop BHR.

One limitation of the study is that from the follow-up examinations no exact data on smoking habits were available for the whole study population. From the present patient material, there was smoking data available only from the follow-up visits of 15 patients from Piirilä et al. (13), three of whom were nonsmokers, five current smokers, and five had stopped smoking before the present studies, basically because of asthma symptoms. The smoking status of them remained similar during the follow-up.

In Finland, patients with suspected asthma are first studied in communal health care centers or hospitals, and they also often have been diagnosed to have asthma by these authorities. If their asthma is suspected to be work-related, it may take even several years before the patient gets to FIOH to be studied if their asthma is work-related. Diagnostics of occupational chemical induced asthma are concentrated to FIOH Helsinki, where the diagnosis is based on specific challenge testing of the chemicals, to which the workers have been exposed to. Because of asthma symptoms or earlier diagnosis of asthma, the patients often have stopped smoking already before they have been examined at FIOH. The patients in the present study had suffered from asthma in mean 2.5 years (SD 3.3 years, range 1–20 years) before the diagnosis of occupational asthma. Therefore, since the patients in this study had long been suffering from respiratory symptoms or had received asthma diagnosis before the occupational studies, it is not very likely that they changed their smoking habits after the diagnosis of occupational asthma, although the possibility remains that some of them may have quitted smoking also after the occupational diagnosis. Even more unlikely is that any of the non-smoking subjects had begun smoking after receiving the occupational diagnosis. Thus, the missing smoking information at the follow-up period is affecting the relative frequencies of smokers and former smokers but not that of non-smokers. From this perspective, it was reasonable to use the smoking status of diagnosis period also in the analysis of follow-up of BHR or FEV%, and we consider that the smoking habits at the occupational diagnosis phase could be a good approximation of the smoking habits also at the control phase.

The relatively small study size also is a limitation of the study, e.g., by restricting us from developing regression models in data calculation because all patients with slow activity genotype were hyperreactive. However, results from the cytokine and IgE analysis as well as use of asthma medication support the observed associations between low activity genotypes and BHR. In addition, there was a slight dominance of females among the study subjects, but as far as we know there has not been suggestion on gender dependence of these associations in the literature.

To summarize, in this study an association between the *GSTP1* slow activity genotype and BHR in DIA patients was found, with suggestion of a simultaneous action of other reduced or normal activity *GST*-genotypes. Also, the Th2 type inflammation was found to be associated with *GSTP1* slow activity genotype. The associations, however, could be recorded only at the follow-up phase when the subjects were not any more exposed to di-isocyanates, and used asthma medication. The results suggest that the presentation of the associations of the low activity enzyme *GST* variants with BHR or asthma may be a dynamic process changing according to treatment, smoking or environmental factors, or may be associated with chronic inflammation. To the best of our knowledge, this is a novel finding, which could offer one explanation for the vast variability of the reported associations in the results found between the lowered activity associated *GST* genotypes and BHR or asthma. We therefore consider that the present results would give important new aspects to address significance of the polymorphic detoxifying enzymes related to BHR or asthma. However, studies with sufficiently large sample sizes are needed to confirm the present findings and to promote our understanding about the complicated interactions between the different enzymes involved in the defense against oxygen radicals in the lungs.

## Ethics Statement

The study was performed according to Helsinki declaration, informed signed consent was received by the participants. The present study received ethic permission form the coordinating ethic committee of the Helsinki and Uusimaa medical district in August 7th, 2012, number 169/13/03/2012. All participants were present or former workers who have diagnosed to have occupational di-isocyanate-índuced asthma, no vulnerable populations were included.

## Author Contributions

JL performed the statistical calculations and reporting and discussion of the results. M-LM performed the analyses of the cytokines and participated the reporting of the results. TS participated in the analyses of the data as well as the reporting of the data. AH participated in the collection of the original data, was responsible for the analysis of genetic data, and participated in the reporting of the results. PP collected and analyzed the original clinical data, organized the analysis of the follow-up of the patients, and as well as reporting of the results.

### Conflict of Interest

The authors declare that the research was conducted in the absence of any commercial or financial relationships that could be construed as a potential conflict of interest. The reviewer HN declared a shared affiliation, with no collaboration, with one of the authors, M-LM, to the handling editor at the time of review.
